# Supplementary material for: Do medical students and residents impact the quality of patient care? An assessment from different stakeholders in an Italian academic hospital, 2019
Source: PLoS One. 2021 Oct 14;16(10):e0258633. doi: 10.1371/journal.pone.0258633 (PMC8516237; doi:10.1371/journal.pone.0258633)
Supplement: S1 Table — (DOCX) [file pone.0258633.s001.docx]

S1 Table. Perception of staff on training by students, residents, patients and HCWs: complete results from generalised ordered logistic regression.

| Quality area | Question  (perception on...) | Staff on training category | Perceived by... | | | | | | | |
| --- | --- | --- | --- | --- | --- | --- | --- | --- | --- | --- |
|  |  |  | Students | | Residents | | Patients | | HCWs | |
|  |  |  | Median (IQR) | OR  (95% CI) | Median (IQR) | OR  (95% CI) | Median (IQR) | OR  (95% CI) | Median (IQR) | OR  (95% CI) |
| A | Assistance improved by staff on training | Residents | 5 (5-5) | 0.78  (0.41 – 1.48) | 5 (5-5) | 1 | 5 (4-5) | 0.49  (0.26 – 0.93) | 4 (3-5) | 0.14  (0.08 – 0.26) |
|  |  | Students | 3 (3-4) | 1 | 3 (2-4) | 0.68  (0.43 – 1.09) | 4 (3-5) | 1. 4.07 (0.53 – 30.96) 2. 7.22 (1.68 – 31.11) 3. 2.94 (1.49 – 5.78) 4. 7.29 (3.45 – 15.37)   * | 3 (2-3) | 0.41  (0.25 – 0.67) |
|  | Human side assistance contribution | Residents | / | / | 4 (3-5) | 1 | / | / | 5 (4-5) | 1.54  (0.79 – 2.98) |
|  |  | Students | 4 (3-4) | 1 | / | / | / | / | 3 (3-4) | 0.42  (0.25 – 0.69) |
| B | Preservation of privacy | Residents | / | / | 5 (4-5) | 1 | 5 (5-5) | 3.17  (1.54 – 6.57) | 5 (4-5) | 0.65  (0.39 – 1.10) |
|  |  | Students | 5 (4-5) | 1 | / | / | 5 (5-5) | 2.40  (1.03 – 5.61) | 4 (3-5) | 0.20  (0.12 – 0.34) |
|  | No accidental data diffusion | Residents | / | / | 5 (4-5) | 1 | 5 (5-5) | 1.74**  (0.56 – 5.40) | 5 (4-5) | 0.36**  (0.16 – 0.80) |
|  |  | Students | 5 (4-5) | 1 | / | / | 5 (4.5-5) | 3.19  (1.50 – 6.79) | 4 (3-5) | 0.39  (0.23 – 0.65) |
| C | Medical record management | Residents | / | / | 5 (4-5) | 1 | / | / | 4 (2-5) | 1. 0.12 (0.15 – 0.90) 2. 0.07 (0.02 – 0.25) 3. 0.09 (0.03 – 0.22) 4. 0.26 (0.15 – 0.44)   * |
|  |  | Students | 5 (5-5) | 1 | / | / | / | / | 4 (3-5) | 0.09  (0.48 – 0.17) |
|  | Informed consent collection | Residents | / | / | 5 (5-5) | 1 | / | / | 5 (4-5) | 0.04**  (0.01 – 0.29) |
|  | Reporting mistakes | Residents | / | / | 4 (4-5) | 1 | / | / | 4 (3-5) | 1. - 2. 0.36 80.12 – 1.15) 3. 0.28 (0.13 – 0.60) 4. 0.66 (0.39 – 1.10)   * |
|  |  | Students | 5 (4-5) | 1 | / | / | / | / | 3 (2-4) | 0.15  (0.08 – 0.27) |
|  | Hand washing | Residents | / | / | 4 (4-5) | 1 | 5 (4-5) | 1. 0.16 (0.02 – 0.98) 2. 0.23 (0.17 – 1.63) 3. 1.39 (0.61 – 3.15) 4. 3.81 (2.13 – 6.83)   * | 4 (4-5) | 0.51  (0.32 – 0.82) |
|  |  | Students | 4 (4-5) | 1 | / | / | 5 (5-5) | 5.12  (2.28 – 11.51) | 4 (3-4) | 0.16  (0.09 – 0.28) |
|  | Patients' pain immediately reported by students | Students | 4 (3-5) | 1 | / | / | 5 (4-5) | 4.07  (1.71 – 9.70) | / | / |
| D | Distinguishing staff on training from HCWs | Residents | / | / | 3 (2-4) | 1 | 4 (2-5) | 1. 0.87 (0.44 – 1.71) 2. 1.74 (1.00 – 3.01) 3. 2.13 (1.25 – 3.63) 4. 3.36 (1.90 – 5.93)   * | 4 (2-4) | 1.20  (0.77 – 1.87) |
|  |  | Students | 4 (2-5) | 1 | / | / | 4 (4-5) | 1. 0.52 (0.13 – 2.00) 2. 4.84 (1.78 – 13.11) 3. 5.43 (2.15 – 13.72) 4. 2.40 (1.22 – 4.74)   * | 4 (3-4) | 1. 0.39 (0.13 – 1.16) 2. 1.68 (0.94 – 3.00) 3. 0.85 (0.51 – 1.44) 4. 0.67 (0.36 – 1.23)   * |
|  | Patients’ trust | Residents | / | / | 4 (4-4) | 1 | 5 (5-5) | 10.2  (5.80 – 18.11) | 4 (3-5) | 1.10  (0.07 – 1.74) |
|  |  | Students | 3 (2-4) | 1 | / | / | 4 (4-5) | 15.23  (7.21 – 32.19) | 3(2-3) | 1. 0.33 (0.15 – 0.72) 2. 1.31 (0.75 – 2.27) 3. 0.77 (0.40 – 1.49) 4. 0.37 (0.08 – 1.73)   * |
|  | Patients' satisfaction of staff on training assistance | Residents | / | / | / | / | 5 (5-5) | 1 | 4 (4-5) | 0.10  (0.05 – 0.18) |
|  |  | Students | / | / | / | / | 4 (3-5) | 1 | 3 (3-4) | 1. 0.70 (0.07 – 6.86) 2. 0.19 (0.04 – 0.84) 3. 0.23 (0.11 – 0.48) 4. 0.07 (0.02 – 0.19)   * |
|  | Adequate students flow within wards | Students | 2 (1-2) | 0.10  (0.05 – 0.19) | 2 (2-3) | 0.19  (0.10 – 0.36) | 4 (2-5) | 1 | 2 (1-3) | 1. 0.10 (0.05 – 0.20) 2. 0.16 (0.08 – 0.33) 3. 0.09 (0.04 – 0.20) 4. 0.05 (0.01 – 0.17)   * |
| *: Generalized ordered logistic regression  ** Logistic regression (1,2,3 scores vs 4,5 scores dichotomy);  /: Question not asked | | | | | | | | | | |
