# Supplementary material for: Do medical students and residents impact the quality of patient care? An assessment from different stakeholders in an Italian academic hospital, 2019
Source: PLoS One. 2021 Oct 14;16(10):e0258633. doi: 10.1371/journal.pone.0258633 (PMC8516237; doi:10.1371/journal.pone.0258633)
Supplement: S1 Appendix — (PDF) [file pone.0258633.s002.pdf]

Questionnaires

Perceived quality of care by patients and healthcare workers concerning staff on training: 2018/2019 assessment

Dear Sir/Madam,

- With the following questionnaire we aim to acquire valuable information regarding your opinion and level of satisfaction with the quality of care provided at Udine Academic Hospital. In particolar, we would like to investigate aspects of patient safety and protection of privacy, quality of care, clinical risk management, ward team, planning of training activities for medical students and postgraduates, by investigating the sensitivity to this issue for each figure involved.

We thank you for your attention and remain at your disposal for any clarification.

EMAIL: smaniotto.cecilia@spes.uniud.it

QUESTIONNAIRE FOR HEALTHCARE WORKERS

(Doctors and nurses)

Unit: drop-down menu

Questions related to RESIDENTS section 1:

1. Are there resident doctors on your ward?
   - ❑ Yes
   - ❑ No

If you answered **NO** to the previous question, go directlyto section 2 –MEDICAL STUDENT

AREA 1- Satisfaction of patients and healthcare workers about the quality of care of staff on training

2. Do patients report satisfaction with the care they receive from residents on the ward? *Please mark only one option.*

   ❑ Total agreement
   ❑ Partial agreement
   ❑ Neutral
   ❑ Partial disagreement
   ❑ Total disagreement
   ❑ No experience/not my concern

   (For subsequent questions where not specified, this is always the answer mode)

3. Do patients readily accept being entrusted to resident doctors?

AREA 2 – Perception of patient safety

4. Do patients feel safe with resident doctors?

5. Do patients ask healthcare workers to check whether the actions and decisions by resident doctors were correct?

AREA 3 –Protection of privacy

6. Do resident doctors protect the patient’s privacy (close the door/let relatives sit outside)?

7. Do resident doctors pay attention to not leaking patient data (loud voice/handovers)?

8. Do resident doctors handle patient documentation with care and put it in the appropriate spaces?

AREA 4 – Quality of care

9. Do resident doctors contribute to the quality of the human relationship with patients?

10. Does the presence of resident doctors increase the quality of care?

11. Are the explanations given by resident doctors sufficient for patients?

12. Do resident doctors collect informed consent from patients?

13. Do resident doctors apply protocols and procedures of the Unit?

AREA 5 –Clinical risk management

14. Do resident doctors wash their hands?

15. Do resident doctors report any adverse event/near misses to staff and/or tutor?

AREA 6 –Perceived role of resident doctors in the team

16. Do patients distinguish resident doctors from staff?

17. Are the staff aware of the resident doctors on the ward?

18. Are resident doctors considered part of the team?

19. Does the training of resident doctors depend on the contribution of all team members?

20. Do resident doctors contribute to a positive atmosphere on the ward?

AREA 7 –General staff satisfaction with the presence of resident doctors

21. Am I satisfied with the quality of the services provided by the resident doctors on the ward?

Questions concerning MEDICAL STUDENTS section 2:

22. Do medical students carry out internship rotations in your ward?
    - ❑ Yes
    - ❑ No

If you answered **NO** to the previous question, go directly to section 3 – PERSONAL DATA

AREA 1- Satisfaction of patients and healthcare workers about the quality of care of staff on training

23. Do patients report satisfaction with the care they receive from medical students on the ward?

24. Are too many medical students medical students in the ward creating discomfort for patients?

25. Do patients readily accept being entrusted to medical students?

AREA 2 - Perception of patient safety

26. Do patients feel safe with medical students?

27. Do patients ask healthcare workers to check whether the actions and decisions by medical students were correct?

AREA 3 –Protection of privacy

28. Do medical students protect the patient’s privacy (close the door/let relatives sit outside)?

29. Do medical students pay attention to not leaking patient data (loud voice/handovers)?

30. Do medical students handle patient documentation with care and put it in the appropriate spaces?

AREA 4 – Quality of care

31. Do medical students contribute to the quality of the human relationship with patients?

32. Does the presence of medical students increase the quality of care?

33. Are the explanations given by medical students sufficient for patients?

AREA 5 –Clinical risk management

34. Do medical students wash their hands?

35. Do medical students report any adverse event/near misses to staff and/or tutor?

AREA 6 - Perceived role of medical students in the team

36. Do patients distinguish medical students from staff?

37. Is the staff aware of the medical students on the ward?

38. Are medical students considered part of the team?

39. Does the training of medical students depend on the contribution of all team members?

40. Do medical students contribute to a positive atmosphere on the ward?

AREA 7 - General staff satisfaction with the presence of medical students

41. Am I satisfied with the quality of the services provided by the medical students on the ward?

Questions concerning PERSONAL DATA section 3:

Gender: ❑ Female❑ Male

Age in years:

Profession:

❑ Doctor

❑ Nurse

QUESTIONNAIRE FOR PATIENTS

UNIT: to be marked when giving the questionnaire to the patient

1. Questions concerning RESIDENT DOCTORS section 1:

Have you ever been examined by a resident doctor?

- ❑ Yes
- ❑ No
- ❑ I don’t know/Not sure

If you answered **NO OR I DON’T KNOW/NOT SURE** to the previous question, go directly to section 2 –MEDICAL STUDENT

AREA 1- Satisfaction of patients and healthcare workers about the quality of care of staff on training

1. Are you satisfied with the care received from residents on the ward?
   Please mark only one option.

   - ❑ Total agreement
   - ❑ Partial agreement
   - ❑ Neutral
   - ❑ Partial disagreement
   - ❑ Total disagreement
   - ❑ No experience/not my concern

   (For subsequent questions where not specified, this is always the answer mode)

2. Are you satisfied with the explanations the resident doctors gave you about your illness?

3. Are you satisfied with how the resident doctors explained the therapies and tests you underwent during your hospital stay?

4. Are you satisfied with the explanations that the resident doctors have given you on what to do when you are discharged and go home?

AREA 2 – Perception of patient safety

5. Do you have trust in the care provided by resident doctors?

AREA 3 –Protection of privacy

6. Are you satisfied with the way resident doctors guarantee your privacy (close the door/let relatives sit outside/manage your data)?

7. Are resident doctors careful that no one hears data/information about patients?

AREA 4 – Quality of care

8. Do the resident doctors explain to you the procedures (i.e. blood sample) they are going to carry out?

9. Does the presence of resident doctors increase the quality of care?

10. When you are interviewed, do resident doctors give you the time you need to explain your situation well?

11. Did the resident doctors give you information that was later corrected/changed by the ward doctor?

AREA 5 – Clinical risk management

12. Do resident doctors wash their hands and/or use hand gel?

- ❑ Always
- ❑ Often
- ❑ Sometimes
- ❑ Rarely
- ❑ Never
- ❑ No experience/not my concern

AREA 6 – Perceived role of resident doctors in the team

13. Can you tell the difference between a resident doctor and a ward doctor?

2. Questions concerning MEDICAL STUDENTS Section 2:

14. Have you ever been examined by a medical student?
    - ❑ Yes
    - ❑ No
    - ❑ I don’t know /not sure

If you answered **NO OR I DONT’ KNOW** to the previous question, go directly to section 3 – PERSONAL DATA

AREA 1- Satisfaction of patients and healthcare workers about the quality of care of staff on training

15. Do you have trust in the care provided by medical students?

16. Are too many medical students in the ward creating discomfort for patients?

AREA 2 – Perception of patient safety

17. Do you feel safe when being assisted by medical students?

AREA 3 – Protection of privacy

18. Are you satisfied with the way medical students guarantee your privacy (close the door/let relatives sit outside/manage your data)?

19. Are medical students careful that no one hears data/information about patients?

AREA 4 – Quality of care

20. Does the presence of medical students increase the quality of care?

21. When patients report pain to medical students, do the students immediately call the nurse or the doctor?

22. Do the medical students sometimes give information which is later corrected/changed by the ward doctor?

AREA 5 – Clinical risk management

23. Do medical students wash their hands and/or use hand gel?

AREA 6 – Perceived role of medical students in the team

24. Can you tell the difference between a medical student and a ward doctor?

25. Please rate on a scale of 1-10 the overall quality of care you received during your hospital stay.

PATIENT PERSONAL DATA SECTION 3:

(NOT the compiler's details)
The questionnaire is compiled:
❑ Directly by the patient
❑ By the patient with the help of a family member or acquaintance
❑ By a family member or acquaintance
❑ By a healthcare professional
Gender: ❑ Female ❑Male

Age in years:

Last school attended: ❑ None ❑Primary school ❑Middle school ❑ Secondary school ❑High school ❑ University degree

QUESTIONNAIRE FOR RESIDENT DOCTORS

AREA 1 - Satisfaction of patients and healthcare workers about the quality of care of staff on training

1. Do patients readily accept being entrusted to resident doctors?
   Please mark only one option.
   - ❑ Total agreement
   - ❑ Partial agreement
   - ❑ Neutral
   - ❑ Partial disagreement
   - ❑ Total disagreement
   - ❑ No experience/not my concern

   (For subsequent questions where not specified, this is always the answer mode)

2. Are too many medical students in the ward creating discomfort for patients?

3. Are too many resident doctors in the ward creating discomfort for patients?

AREA 2 – Perception of patient safety

4. Do patients feel safe when being entrusted to resident doctors?

5. Do patients trust resident doctors when they need explanations?
6. Do patients undergo certain procedures irrespective of whether they are carried out by a resident doctor or a ward physician?

AREA 3 – Protection of privacy

7. Are patients worried about resident doctors knowing their data?

8. Do resident doctors always guarantee patient privacy (close the door/let the relatives sit outside)?

9. Are resident doctors careful that no one hears patient data/information?

10. Do resident doctors store patient documentation in the appropriate spaces?

AREA 4 – Quality of care

11. Do resident doctors give useful instructions for the patient when he/she goes home?
12. Does the presence of resident doctors increase the quality of care?

13. Does the presence of medical students increase the quality of care?

14. Do resident doctors contribute to the quality of the human relationship with patients?

15. Do ward doctors give the same information to patients as what previously given by resident doctors?

16. Do resident doctors collect the informed consent from the patient before a procedure/intervention?

17. Do resident doctors pay attention to patients in order to readily detect if they have pain?

18. Do resident doctors only carry out procedures in which they feel competent?

19. Do resident doctors apply protocols and procedures of the Unit?

AREA 5 – Clinical risk management

20. Do resident doctors wash their hands at the five key moments identified by WHO (i.e. before patient contact, before an aseptic procedure, after an aseptic procedure, after patient contact, after contact with objects around the patient)?

21. If resident doctors make mistakes, do they report them immediately to the tutor and/or staff?

AREA 6 – Perceived role of resident doctors in the team

22. Do patients distinguish resident doctors from staff?

23. Do resident doctors always have to wear a name tag to be recognised by patients?

24. Do the staff know who are the resident doctors in their ward/department?

25. Are resident doctors considered part of the ward team?

26. Does the training of resident doctors depend on the contribution of all team members?

27. Do resident doctors improve the atmosphere in the ward?

RESIDENT DOCTOR PERSONAL DATA:

Gender: ❑ Female ❑ Male
Age in years:
Residency:
Year of attendance:
❑ I ❑ II ❑ III ❑ IV ❑ V ❑VI

QUESTIONNAIRE FOR MEDICAL STUDENTS

AREA 1- Satisfaction of patients and healthcare workers about the quality of care of staff on training

1. Do patients readily accept being entrusted to medical students?

Please mark only one option.

- ❑ Total agreement
- ❑ Partial agreement
- ❑ Neutral
- ❑ Partial disagreement
- ❑ Total disagreement
- ❑ No experience/not my concern

(For subsequent questions where not specified, this is always the answer mode)

2. Are too many medical students in the ward creating discomfort for patients?

AREA 2 – Perception of patient safety

3. Do patients feel safe when being entrusted to medical students?

AREA 3 – Protection of privacy

4. Are patients worried about medical students knowing their data?

5. Do medical students always guarantee patient privacy (close the door/let the relatives sit outside)?

6. Are medical students careful that no one hears patient data/information?

7. Do medical students store patient documentation in the appropriate spaces?

AREA 4 – Quality of care

8. Does the presence of medical students increase the quality of care?

9. Does the presence of resident doctors increase the quality of care?

10. Do medical students contribute to the quality of the human relationship with patients?

11. Do medical students pay attention to patients in order to readily detect if they have pain?

12. Do medical students only carry out procedures in which they feel competent?

AREA 5 – Clinical risk management

13. Do medical students wash their hands at the five key moments identified by WHO (i.e. before patient contact, before an aseptic procedure, after an aseptic procedure, after patient contact, after contact with objects around the patient)?

14. If medical students make mistakes, do they report them immediately to the tutor and/or staff?

AREA 6 – Perceived role of medical students in the team

15. Do patients distinguish medical students from staff?

16. Do medical students always have to wear a name tag to be recognised by patients?

17. Do the staff know who are the medical students in their ward/department?

18. Are medical students considered part of the ward team?

19. Does the training of medical students depend on the contribution of all team members?

20. Do medical students improve the atmosphere in the ward?

21. Please rate on a scale of 1-10 the overall quality of the training in the last unit/department you attended.

MEDICAL STUDENT PERSONAL DATA:

Gender: ❑ Female ❑ Male
Age in years:
Year of attendance:
❑ IV ❑ V ❑VI
Last unit/department attended:
