# Supplementary material for: Do medical students and residents impact the quality of patient care? An assessment from different stakeholders in an Italian academic hospital, 2019
Source: PLoS One. 2021 Oct 14;16(10):e0258633. doi: 10.1371/journal.pone.0258633 (PMC8516237; doi:10.1371/journal.pone.0258633)
Supplement: S2 Appendix — (PDF) [file pone.0258633.s003.pdf]

Questionari

Soddisfazione dei pazienti e degli operatori in relazione al personale in formazione: valutazione 2018/2019

Gent. ma/o,

- Con il seguente questionario ci proponiamo di acquisire preziose informazioni inerenti la Sua opinione e il suo livello di soddisfazione circa la qualità dell'assistenza erogata all'interno dell'Azienda Sanitaria Universitaria Integrata di Udine. Desideriamo in particolare approfondire aspetti di sicurezza del paziente e protezione della privacy, qualità delle cure erogate, gestione del rischio clinico, composizione del team di reparto, pianificazione delle attività formative per studenti e specializzandi, indagando la sensibilità a questa tematica di ogni figura coinvolta.

La ringraziamo per la Sua attenzione e restiamo a disposizione per qualsiasi chiarimento.

EMAIL: smaniotto.cecilia@spes.uniud.it

QUESTIONARIO RIVOLTO AI PROFESSIONISTI SANITARI

(Dirigenti medici ed infermieri)

Unità operativa: menu a tendina

Domande relative ai MEDICI SPECIALIZZANDI sezione 1:

1. Nel tuo reparto prestano servizio medici specializzandi?
   - ❑ Si
   - ❑ No

Se hai risposto **NO** alla precedente domanda vai direttamente alla sezione 2 -STUDENTE DI MEDICINA

AREA 1- Soddisfazione di pazienti e operatori sulla qualità dell'assistenza del personale in formazione

2. I pazienti riferiscono soddisfazione rispetto all'assistenza ricevuta dai medici specializzandi nel reparto?
   Contrassegna solo un'opzione.

   - ❑ Totale accordo
   - ❑ Parziale accordo
   - ❑ Neutrale
   - ❑ Parziale disaccordo
   - ❑ Totale disaccordo
   - ❑ Non ho esperienza/non mi riguarda

   (Per le domande successive dove non specificato si intende sempre questa modalità di risposta)
3. I pazienti accettano senza problemi di essere affidati ai medici specializzandi?

AREA 2 - Percezione di sicurezza dei pazienti

4. I pazienti si sentono sicuri nelle mani dei medici specializzandi?

5. I pazienti chiedono al professionista strutturato di controllare quanto appena fatto di medici specializzandi?

AREA 3 - Tutela della privacy

6. I medici specializzandi tutelano la privacy del paziente (chiudono la porta/ fanno accomodare fuori i parenti)?

7. I medici specializzandi prestano attenzione a non far trapelare i dati del paziente (voce alta / scambio delle consegne)?

8. I medici specializzandi gestiscono con cura la documentazione dei pazienti riponendola negli appositi spazi?

AREA 4 - Qualità dell'assistenza fornita

9. I medici specializzandi contribuiscono alla qualità della relazione umana col paziente?

10. La presenza dei medici specializzandi aumenta la qualità dell'assistenza?

11. Per i pazienti sono sufficienti le spiegazioni fornite dai medici specializzandi?

12. I medici specializzandi raccolgono il consenso informato dai pazienti?

13. I medici specializzandi applicano protocolli e procedure della UO?

AREA 5 - Gestione del rischio clinico

14. I medici specializzandi si lavano le mani?

15. I medici specializzandi riferiscono eventuali eventi avversi /quasi eventi / errori al personale e/o al tutor?

AREA 6 - Ruolo percepito dei medici specializzandi nella équipe

16. I pazienti distinguono i medici specializzandi dal personale?

17. Il personale è al corrente dei medici specializzandi che ci sono in reparto?

18. I medici specializzandi sono considerati parte dell'equipe?

19. La formazione dei medici specializzandi dipende dal contributo di tutti i membri dell'equipe?

20. I medici specializzandi contribuiscono a creare un clima positivo in reparto?

AREA 7 - Soddisfazione generale del personale nei confronti della presenza dei medici specializzandi

21. Sono soddisfatto della qualità delle prestazioni erogate dai medici specializzandi in reparto?

Domande relative agli STUDENTI MEDICI sezione 2:

22. Nel tuo reparto effettuano rotazioni di tirocinio studenti medici?
    - ❑ Sì
    - ❑ No

Se hai risposto **NO** alla precedente domanda vai direttamente alla sezione 3 – ANAGRAFICA

AREA 1- Soddisfazione di pazienti e operatori sulla qualità dell'assistenza del personale in formazione

23. I pazienti riferiscono soddisfazione rispetto alla presenza degli studenti di medicina nel reparto?

24. Troppi studenti di medicina nel reparto creano disagio ai pazienti?

25. I pazienti accettano senza problemi di essere affidati agli studenti di medicina?

AREA 2 - Percezione di sicurezza dei pazienti

26. I pazienti si sentono sicuri nelle mani degli studenti di medicina?

27. I pazienti chiedono al professionista di controllare quanto appena fatto dagli studenti di medicina?

AREA 3 - Tutela della privacy

28. Gli studenti di medicina tutelano la privacy del paziente (chiudono la porta/ fanno accomodare fuori i parenti)?

29. Gli studenti di medicina prestano attenzione a non far trapelare i dati del paziente (voce alta/ scambio delle consegne)?

30. Gli studenti di medicina gestiscono con cura la documentazione dei pazienti riponendola negli appositi spazi?

AREA 4 - Qualità dell'assistenza fornita

31. Gli studenti di medicina contribuiscono alla qualità della relazione umana col paziente?

32. La presenza degli studenti di medicina aumenta la qualità dell'assistenza?

33. Per i pazienti sono sufficienti le spiegazioni fornite dagli studenti di medicina?

AREA 5 - Gestione del rischio clinico

34. Gli studenti di medicina si lavano le mani?

35. Gli studenti di medicina riferiscono eventuali eventi avversi/ quasi eventi/ errori al personale e/o al tutor?

AREA 6 - Ruolo percepito degli studenti di medicina nella équipe

36. I pazienti distinguono gli studenti di medicina dal personale?

37. Il personale è al corrente degli studenti di medicina che ci sono in reparto?

38. Gli studenti di medicina sono considerati parte dell'equipe?

39. La formazione degli studenti di medicina dipende dal contributo di tutti i membri dell'equipe?

40. Gli studenti di medicina contribuiscono a creare un clima positivo in reparto?

AREA 7 - Soddisfazione generale del personale nei confronti della presenza degli studenti di medicina

41. Sono soddisfatto della qualità delle prestazioni erogate dagli studenti di medicina in reparto?

Domande relative all'ANAGRAFICA sezione 3:

Sesso: ❑ Femmina ❑ Maschio
Età in anni compiuti:
Professione:
❑ Medico
❑ Infermiere

QUESTIONARIO RIVOLTO AI PAZIENTI

REPARTO: da segnare quando si consegna il questionario al paziente

1. Domande relative ai MEDICI SPECIALIZZANDI sezione 1:

Sei mai stato visitato da un medico specializzando?

- ❑ Si
- ❑ No
- ❑ Non so /non ne sono sicuro

Se hai risposto **NO O NON SO/NON NE SONO SICURO** alla precedente domanda vai direttamente alla sezione 2 - STUDENTE DI MEDICINA

AREA 1- Soddisfazione di pazienti e operatori sulla qualità dell'assistenza del personale in formazione

1. Sei soddisfatto dell'assistenza dei medici specializzandi?
   Contrassegna solo un'opzione.

   - ❑ Totale accordo
   - ❑ Parziale accordo
   - ❑ Neutrale
   - ❑ Parziale disaccordo
   - ❑ Totale disaccordo
   - ❑ Non ho esperienza/non mi riguarda

   (per le domande successive, dove non specificato, si intende sempre questa modalità di risposta)

2. Sei soddisfatto delle spiegazioni che i medici specializzandi ti hanno dato sulla tua malattia?

3. Sei soddisfatto di come i medici specializzandi ti hanno spiegato le terapie e gli esami che hai effettuato durante il ricovero?

4. Sei soddisfatto delle spiegazioni che i medici specializzandi ti hanno dato su cosa fare quando sarai dimesso e andrai a casa?

AREA 2 - Percezione di sicurezza dei pazienti

5. Riponi fiducia nell'assistenza prestata dai medici specializzandi?

AREA 3 - Tutela della privacy

6. Sei soddisfatto di come i medici specializzandi garantiscono la tua privacy (chiudono la porta / fanno accomodare fuori i parenti/gestiscono i tuoi dati)?

7. I medici specializzandi sono attenti che nessuno senta i dati / le informazioni che riguardano i pazienti?

AREA 4 - Qualità dell’assistenza fornita

8. I medici specializzandi ti spiegano le procedure (es. prelievi) che stanno per effettuare?

9. La presenza dei medici specializzandi aumenta la qualità dell’assistenza?

10. Quando ti intervistano, i medici specializzandi ti lasciano tutto il tempo necessario per spiegare bene la tua situazione?

11. È capitato che i medici specializzandi ti abbiano dato informazioni che dopo sono state corrette / modificate dal medico di reparto?

AREA 5 - Gestione del rischio clinico

12. I medici specializzandi si lavano le mani e/o usano il gel per le mani?

   - ❑ Sempre
   - ❑ Spesso
   - ❑ Qualche volta
   - ❑ Raramente
   - ❑ Mai
   - ❑ Non ho esperienza/non mi riguarda

AREA 6 - Ruolo percepito dei medici specializzandi nella équipe

13. Riesci a distinguere un medico specializzando da un medico strutturato del reparto?

PARTE STUDENTE DI MEDICINA SEZIONE 2:

14. Sei mai stato visitato da uno studente di medicina?
   - ❑ Si
   - ❑ No
   - ❑ Non so /non ne sono sicuro

Se ha risposto **NO O NON SO** alla precedente domanda vada direttamente alla sezione 3 – ANAGRAFICA

AREA 1- Soddisfazione di pazienti e operatori sulla qualità dell’assistenza del personale in formazione

15. Riponi fiducia nell'assistenza prestata dagli studenti di medicina?

16. Troppi studenti di medicina nel reparto creano disagio ai pazienti?

AREA 2 - Percezione di sicurezza dei pazienti

17. Ti senti sicuro quando ti assistono gli studenti di medicina?

AREA 3 - Tutela della privacy

18. Sei soddisfatto di come gli studenti di medicina garantiscono la tua privacy (chiudono la porta / fanno accomodare fuori i parenti/gestiscono i tuoi dati)?

19. Gli studenti di medicina sono attenti che nessuno senta i dati / le informazioni che riguardano i pazienti?

AREA 4 - Qualità dell'assistenza fornita

20. La presenza degli studenti di medicina aumenta la qualità dell'assistenza?

21. Quando i pazienti riferiscono agli studenti di medicina di avere dolore, gli studenti chiamano subito l'infermiere o il medico?

22. Capita che gli studenti di medicina diano informazioni che dopo vengono corrette / modificate dal medico del reparto?

AREA 5 - Gestione del rischio clinico

23. Gli studenti di medicina si lavano le mani e/o usano il gel per le mani?

AREA 6 - Ruolo percepito degli studenti di medicina nella équipe

24. Riesci a distinguere uno studente di medicina da unmedico specializzando del reparto?

25. Valuta da 1 a 10 la qualità generale dell'assistenza ricevuta durante il tuo ricovero.

PARTE ANAGRAFICA PAZIENTE SEZIONE 3:

(NON l'anagrafica del compilatore)
Il questionario è compilato:
❑ Direttamente dal paziente
❑ Dal paziente con l'aiuto di un familiare o conoscente
❑ Da un familiare o conoscente
❑ Da un operatore sanitario
Sesso: ❑ Femmina ❑ Maschio

Età in anni compiuti:

Ultima scuola frequentata: ❑ Nessuno ❑ Elementari ❑ Media Inferiore ❑ Media ❑Superiore ❑ Titolo Universitario

QUESTIONARIO RIVOLTO AGLI SPECIALIZZANDI

AREA 1 - Soddisfazione di pazienti e operatori sulla qualità dell'assistenza del personale in formazione

1. I pazienti accettano senza problemi di essere affidati ai medici specializzandi?
   Contrassegna solo un'opzione.
   ❑ Totale accordo
   ❑ Parziale accordo
   ❑ Neutrale
   ❑ Parziale disaccordo
   ❑ Totale disaccordo
   ❑ Non ho esperienza/non mi riguarda

   (per le domande successive, dove non specificato, si intende sempre questa modalità di risposta)

2. Troppi studenti di medicina nel reparto creano disagio ai pazienti?

3. Troppi medici specializzandi nel reparto creano disagio ai pazienti?

AREA 2 - Percezione di sicurezza dei pazienti

4. I pazienti si sentono sicuri nelle mani dei medici specializzandi?

5. I pazienti si rivolgono ai medici specializzandi se necessitano di spiegazioni?
6. I pazienti si sottopongono a determinate prestazioni indipendentemente dal fatto che queste siano effettuate da un medico specializzando o da un medico strutturato del reparto?

AREA 3 - Tutela della privacy

7. I pazienti sono preoccupati che i medici specializzandi conoscano i loro dati?

8. I medici specializzandi garantiscono sempre la privacy del paziente (chiudono la porta / fanno accomodare fuori i parenti)?

9. I medici specializzandi sono attenti che nessuno senta i dati / le informazioni dei pazienti?

10. I medici specializzandi ripongono negli appositi spazi la documentazione del paziente?

AREA 4 - Qualità dell'assistenza fornita

11. I medici specializzandi danno istruzioni utili al paziente per quando andrà a casa?
12. La presenza dei medici specializzandi aumenta la qualità dell'assistenza?

13. La presenza degli studenti di medicina aumenta la qualità dell'assistenza?

14. I medici specializzandi contribuiscono alla qualità della relazione umana col paziente?

15. I medici strutturati danno ai pazienti le stesse informazioni rispetto a quelle date in precedenza dai medici specializzandi?

16. I medici specializzandi fanno firmare al paziente il consenso prima di un esame/intervento?

17. I medici specializzandi prestano attenzione ai pazienti per cogliere tempestivamente se i pazienti hanno dolore?

18. I medici specializzandi effettuano solo prestazioni in cui si sentono competenti?

19. I medici specializzandi applicano protocolli e procedure della UO?

AREA 5 - Gestione del rischio clinico

20. I medici specializzandi si lavano le mani nei cinque momenti fondamentali individuati dall’OMS (ovvero prima del contatto con il paziente, prima di una manovra asettica, dopo esposizione ad un liquido biologico, dopo il contatto con il paziente, dopo il contatto con ciò che sta attorno al paziente)?

21. Se i medici specializzandi fanno errori, li riferiscono subito al tutor e/o al personale?

AREA 6 - Ruolo percepito degli studenti di medicina nella équipe

22. I pazienti sanno distinguere i medici specializzandi dai medici strutturati del reparto?

23. I medici specializzandi devono portare sempre il cartellino per farsi riconoscere dai pazienti?

24. Il personale sa chi sono i medici specializzandi all’interno del proprio reparto?

25. I medici specializzandi vengono considerati parte dell’equipe?

26. La formazione dei medici specializzandi dipende dal contributo di tutti i membri dell’equipe?

27. I medici specializzandi migliorano il clima di reparto?

PARTE ANAGRAFICA MEDICO SPECIALIZZANDO:

Sesso: ❑ Femmina ❑ Maschio
Età in anni compiuti:
Scuola di specializzazione:
Anno di corso:
❑ I ❑ II ❑ III ❑ IV ❑ V ❑ VI

QUESTIONARIO RIVOLTO AGLI STUDENTI DI MEDICINA

AREA 1- Soddisfazione di pazienti e operatori sulla qualità dell'assistenza del personale in formazione

1. I pazienti accettano senza problemi di essere affidati agli studenti di medicina?

Contrassegna solo un'opzione.

- ❑ Totale accordo
- ❑ Parziale accordo
- ❑ Neutrale
- ❑ Parziale disaccordo
- ❑ Totale disaccordo
- ❑ Non ho esperienza/non mi riguarda

(per le domande successive, dove non specificato, si intende sempre questa modalità di risposta)

2. Troppi studenti di medicina nel reparto creano disagio ai pazienti?

AREA 2 - Percezione di sicurezza dei pazienti

3. I pazienti si sentono sicuri nelle mani degli studenti di medicina?

AREA 3 - Tutela della privacy

4. I pazienti sono preoccupati che gli studenti di medicina conoscano i loro dati?

5. Gli studenti di medicina garantiscono sempre la privacy del paziente (chiudono la porta / fanno accomodare fuori i parenti)?

6. Gli studenti di medicina sono attenti che nessuno senta i dati / le informazioni dei pazienti?

7. Gli studenti di medicina ripongono negli appositi spazi la documentazione del paziente?

AREA 4 - Qualità dell'assistenza fornita

8. La presenza degli studenti di medicina aumenta la qualità dell'assistenza?

9. La presenza dei medici specializzandi aumenta la qualità dell'assistenza?

10. Gli studenti di medicina contribuiscono alla qualità della relazione umana col paziente?

11. Gli studenti di medicina prestano attenzione ai pazienti per cogliere tempestivamente se i pazienti hanno dolore?

12. Gli studenti di medicina effettuano solo prestazioni in cui si sentono competenti?

AREA 5 - Gestione del rischio clinico

13. Gli studenti di medicina si lavano le mani nei cinque momenti fondamentali dell'OMS?

(Ovvero prima del contatto con il paziente, prima di una manovra asettica, dopo esposizione ad un liquido biologico, dopo il contatto con il paziente, dopo il contatto con ciò che sta attorno al paziente)

14. Se gli studenti di medicina fanno errori, li riferiscono subito al tutor e/o al personale?

AREA 6 - Ruolo percepito degli studenti di medicina nella équipe

15. I pazienti sanno distinguere gli studenti di medicina dai medici del reparto?

16. Gli studenti medici devono portare sempre il cartellino per farsi riconoscere dai pazienti?

17. Il personale sa chi sono gli studenti di medicina all'interno del proprio reparto?

18. Gli studenti di medicina vengono considerati parte dell'equipe?

19. La formazione degli studenti di medicina dipende dal contributo di tutti i membri dell'equipe?

20. Gli studenti di medicina migliorano il clima di reparto?

21. Valuta da 1 a 10 la qualità del tirocinio nell'ultimo reparto da te frequentato.

PARTE ANAGRAFICA STUDENTE DI MEDICINA SEZIONE 3:

Sesso: ❑ Femmina ❑ Maschio
Età in anni compiuti:
Anno di corso:
❑ IV ❑ V ❑ VI
Ultimo reparto frequentato:
