# Supplementary material for: Do medical students and residents impact the quality of patient care? An assessment from different stakeholders in an Italian academic hospital, 2019
Source: PLoS One. 2021 Oct 14;16(10):e0258633. doi: 10.1371/journal.pone.0258633 (PMC8516237; doi:10.1371/journal.pone.0258633)
Supplement: Attachment — Submitted filename: Response to reviewers.docx [file pone.0258633.s005.docx]

Udine, July 29^th^, 2021

To the kind attention of dr. Sharon Mary Brownie (Academic Editor, *Plos One*),

Please find here enclosed the revised version of the manuscript entitled “Does staff on training have an impact on quality of care? An assessment from different stakeholders in an Italian academic hospital, 2019.” by Perri G, d’Angelo M, Smaniotto C, Del Pin M, Ruscio E, Londero C, Brunelli L, Castriotta L, Brusaferro S.

The manuscript now includes the alterations required by Reviewer 1 and Reviewer 2 to better present the research. More specifically, the manuscript has been modified as follows:

a) Reviewer 1:

1) The study design is now more specifically described in the Methods section.

2) The number of collected questionnaires and interviewed subjects is now correct.

3) Data analysis was completed with the explanation and a new reference for the assumptions to use generalised ordered logistic regression.

b) Reviewer 2:

1) The background section has been shortened.

2) A quantitative method of data collection coupled with the qualitative one could be used in the next edition of the study. For the study carried out in 2019, the method used in the pilot study of 2017 was kept.

3) Line 29: “Respectively” has been removed..

4) The number of collected questionnaires and interviewed subjects is now correct.

5) The title has been modified from “What patients, healthcare workers, residents and students think about quality of care in an Italian academic hospital?” to “Does staff on training have an impact on quality of care? An assessment from different stakeholders in an Italian academic hospital, 2019.” to better match the main objective.

6) The explanation of main objective and secondary objectives has been modified in order to have a better concordance with both title and main text.

7) Refusing to participate to the study has been removed from the exclusion criteria as being willing to participate is already an inclusion criterion.

8) Conclusions have been modified in order to have a better concordance with results and discussion.

9) Expected response rate for patients was lower than expected response rate for the other stakeholder categories as inpatiens could be unable to partecipate albeit willing, due to their physical or psychological conditions at the moment of the questionnaire collection. Assuming a 70% expected responde rate for inpatients, the minimum number of needed questionnaires would have been 66.

10) Line 175-179: the number of collected questionnaires and interviewed subjects is now correct.

11) Line 269: non-respondent rate is now mentioned.

12) When referring to both categories together, “staff on training” is now used to describe “medical students and residents”.

The minimum anonymised data set to replicate the study is now available as a Supporting Information file. Four Supporting Information files are now available, including two appendixes for questionnaires (Italian and English version). A new table presents the complete results from generalised ordered logistic regression

All authors have read and approved the manuscript. The paper has not been published, and is not under review elsewhere. There are no ethical problems or conflicts of interest.

We thank you in advance for your kind consideration.

With kindest regards

Sincerely Yours,

Cecilia Smaniotto, MD

Corresponding author

Department of Medicine, University of Udine.

Address: Via Colugna 50, 33100 Udine, Italy.

Phone +390432554767. Email: [smaniotto.cecilia@spes.uniud.it](mailto:smaniotto.cecilia@spes.uniud.it) smaniotto.cecilia@spes.uniud.it
