# Supplementary material for: Do medical students and residents impact the quality of patient care? An assessment from different stakeholders in an Italian academic hospital, 2019
Source: PLoS One. 2021 Oct 14;16(10):e0258633. doi: 10.1371/journal.pone.0258633 (PMC8516237; doi:10.1371/journal.pone.0258633)
Supplement: Attachment — Submitted filename: Response to reviewers (30.09.2021).docx [file pone.0258633.s006.docx]

Udine, September 28^th^, 2021

To the kind attention of dr. Sharon Mary Brownie (Academic Editor, *Plos One*),

Please find here enclosed the revised version of the manuscript entitled “Does staff on training have an impact on quality of care? An assessment from different stakeholders in an Italian academic hospital, 2019.” by Perri G, d’Angelo M, Smaniotto C, Del Pin M, Ruscio E, Londero C, Brunelli L, Castriotta L, Brusaferro S. It is now entitled “Do medical students and residents impact the quality of patient care? An assessment from different stakeholders in an Italian academic hospital, 2019.”

The manuscript now includes the alterations required by Reviewer 3 to better present the research. The manuscript has been modified as follows:

1) The results referred to Table 2 are now more clearly highlighted in the Results section.
2) The errors in line 185-193 have been corrected.
3) More subtitles have been added in the methods section (*Design of the study and previous research*; *Design of the questionnaire and testing phase*; *Inclusion and exclusion criteria*; *Data collection*).
4) The title has been revised as suggested. Nonetheless, the previous reference to “medical students and residents” has been kept as this was the suggestion of Reviewer 2 in Revision 1, and it was applied throughout the text. The authors can change again the reference back to “staff on training”, however, if this would improve clarity and readability of the article.

All authors have read and approved the manuscript. The paper has not been published, and is not under review elsewhere. There are no ethical problems or conflicts of interest.

We thank you in advance for your kind consideration.

With kindest regards

Sincerely Yours,

Cecilia Smaniotto, MD

Corresponding author

Department of Medicine, University of Udine.

Address: Via Colugna 50, 33100 Udine, Italy.

Phone +390432554767. Email: [smaniotto.cecilia@spes.uniud.it](mailto:smaniotto.cecilia@spes.uniud.it) smaniotto.cecilia@spes.uniud.it
